# Stem Cells as a Tool to Improve Outcomes of Islet Transplantation

**DOI:** 10.1155/2012/736491

**Published:** 2012-08-30

**Authors:** Emily Sims, Carmella Evans-Molina

**Affiliations:** ^1^Section of Pediatric Endocrinology and Diabetology, Department of Pediatrics, Indiana University School of Medicine, Indianapolis, IN 46202, USA; ^2^Section of Endocrinology and Metabolism, Department of Medicine, Indiana University School of Medicine, Indianapolis, IN 46202, USA; ^3^Department of Cellular and Integrative Physiology, Indiana University School of Medicine, Indianapolis, IN 46202, USA; ^4^The Herman B Wells Center for Pediatric Research, Indiana University School of Medicine, Indianapolis, IN 46202, USA

## Abstract

The publication of the promising results of the Edmonton protocol in 2000 generated optimism for islet transplantation as a potential cure for Type 1 Diabetes Mellitus. Unfortunately, follow-up data revealed that less than 10% of patients achieved long-term insulin independence. More recent data from other large trials like the Collaborative Islet Transplant Registry show incremental improvement with 44% of islet transplant recipients maintaining insulin independence at three years of follow-up. Multiple underlying issues have been identified that contribute to islet graft failure, and newer research has attempted to address these problems. Stem cells have been utilized not only as a functional replacement for **β** cells, but also as companion or supportive cells to address a variety of different obstacles that prevent ideal graft viability and function. In this paper, we outline the manners in which stem cells have been applied to address barriers to the achievement of long-term insulin independence following islet transplantation.

## 1. Introduction: An Emerging Field in Treatment for Type I Diabetes Mellitus

The promising results of the Edmonton protocol, published in 2000, brought new enthusiasm to the field of islet transplantation. With this method, Shapiro et al. combined a glucocorticoid-free immunosuppression regimen with improved techniques for islet isolation and purification, followed by transplantation via percutaneous transhepatic portal embolization of 4000 islet equivalents per kilogram of body weight. Although most patients required repeated transplants to achieve insulin independence, at a median follow-up of 11.9 months, all seven patients who had undergone islet transplantation had no requirements for exogenous insulin. Prior to the procedure, recipients uniformly suffered from recurrent severe hypoglycemic episodes, and they experienced resolution of these episodes with increased stability in blood glucose values afterwards. No major complications occurred. These outcomes generated hope that islet transplantation would optimize metabolic control in patients with Type 1 Diabetes Mellitus (T1DM) and obviate the need for exogenous insulin administration [1]. 

However, to the great disappointment of the medical and research communities, long-term follow-up of transplanted patients revealed less encouraging outcomes. Among a cohort of 65 patients followed for an average of 35.5 months after transplantation, most were able to achieve short-term insulin independence, but 92.5% eventually required insulin to maintain glycemic control. These results were tempered by persistent C-peptide positivity in 82% of patients, as well as sustained improvements in hypoglycemia and blood glucose lability in those with surviving grafts [2]. The recently released report of the Collaborative Islet Transplant Registry is more encouraging with 44% of the 208 allograft recipients in the 2007–2010 era maintaining insulin independence at 3 years of follow-up, but still leaves room for improvement [3]. These limitations of long-term graft function have brought attention to the need for further refinements in the islet isolation and transplantation process. 

## 2. Etiologies of Graft Dysfunction

Multiple reasons have been cited as underlying etiologies of graft failure. The period surrounding transplantation is marked by a rapid loss of approximately 50–70% of donor islets [4, 5]. This large-scale islet loss is partly caused by the quality of donor pancreata as well as the isolation process itself, which includes enzymatic digestion, cold-storage time, and exposure to hypoxia during isolation and culture [6]. These combined stresses generate inflammatory cytokines and initiate proapoptotic NF-*κ*B, mitogen-activated kinase, and poly(ADP-ribose) polymerase stress pathways before transplantation has even occurred [7, 8].

Another major culprit in early islet death is the innate immune system, which launches a large-scale inflammatory reaction, initiating massive *β* cell death immediately after percutaneous infusion [4, 9]. This instant blood-mediated inflammatory reaction (IBMIR) occurs secondary to the brisk activation of coagulation and complement cascades, which are triggered by exposure to human blood [10]. While collagen residues and islet surface molecules activate the intrinsic coagulation cascade, tissue factor, which is secreted by transplanted islets and expressed on ductal cells that contaminate islet preparations, activates the extrinsic cascade. Coagulation as well as collagen residues lead to platelet activation. Complement activation can occur also via classical and alternative pathways. These processes are followed by spread of inflammatory cells into the islet, resulting in cytotoxicity and the eventual progression down apoptotic pathways [9–11]. 

Although the innate immune response causes early *β* cell death, long-term deterioration in graft function is also related to allo- and autoimmunity [2]. Similar to solid organ transplants, islet grafts are susceptible to the development of allograft rejection via sensitization to alloantigens presented by antigen presenting cells and subsequent activation of a T cell-dependent immune response [12, 13]. Unfortunately, another consequence of the IBMIR is antigen presentation of transplanted islets by infiltrating neutrophils and macrophages [11]. The rejection process can be mitigated by immunosuppressants, but once drug levels are decreased, allosensitization occurs [12]. Alloreactive T cell activity, measured by presence of cytotoxic T cell precursors, is strongly associated with graft failure, although this influence can also be affected by the type of immunosuppression regimen [14, 15]. The similarity in function and viability between autografts and allografts containing twice as many islets underscores the role of alloreactivity in underperformance of allografts [16]. 

Given that T1DM is an autoimmune disease, recurrent autoimmune destruction involving donor islet antigens may also play a role in graft failure. Monocytic infiltration of islet grafts with preferential *β* cell loss has been demonstrated weeks after transplant [17]. Studies evaluating autoantibody influence on transplanted islet success have shown varying associations. The autoimmune process appears to largely contribute to graft destruction in the context of a donor and recipient MHC class II antigen match [18]. While also likely affected by differences in preparation and immunosuppressive regimens, higher baseline lymphocyte counts, and T cell autoreactivity against islet-associated antigens are negatively associated with graft function [14, 19–21]. 

Another hurdle that has emerged as a limitation in graft viability and function is the development of an optimal vascular network [22]. Normal pancreatic islets have an extensive microvascular system. Capillaries supplying the endocrine cells are more numerous, with thinner walls, more extensive fenestrations, and larger diameters than exocrine components, suggesting an increased importance of perfusion and sensitivity to hypoxia [23]. Unfortunately, this network is interrupted during the islet isolation process [22]. Transplanted islet oxygen, nutrient supply, and exposure to intraislet paracrine signaling are limited by rate of neovascularization and alterations in the vascular development that differ compared to the vascular networks seen in native islets [24, 25].

The site of transplantation may also have important implications. Aside from islet exposure to the IBMIR, the portal vein has several drawbacks as a transplant site [25]. Islets may be exposed to higher concentrations of *β* cell toxic immunosuppressants via the portal vein [26]. Oxygen concentrations supplied by the portal vein are lower than those from arterial supplies, resulting in relative hypoxia of the islet graft. Pancreatic islets transplanted intraportally into the liver of mice also have substantially lower blood flow than native islets [25, 27]. Because of glycogen and glucose production by the liver, vascular communication with surrounding hepatocytes can expose transplanted islets to higher glucose concentrations than seen in the systemic circulation. This results in impairment of the appropriate response of *β* cells and *α* cells to systemic blood glucose levels [28, 29]. Development of hepatic steatosis may also negatively affect graft function [30]. 

## 3. Stem Cells as a Tool to Address Limitations of Islet Transplants

Recent advances in the field of stem cell research have stimulated significant interest in the potential role that both multipotent (adult), pluripotent (embryonic), and induced pluripotent stem cells could play in the replacement of islets in patients with T1DM. The unique properties of different postnatal or adult stem cell populations offer valuable supportive functions that appear to enhance graft function and survival. In addition to a potential role as companion cells during transplantation, pluripotent or embryonic stem cells present a potential alternative for the generation of insulin producing cells (IPCs). The use of adult stem cell populations has also emerged as a potential source of IPCs, and these cells can be directed down a pancreatic and endocrine lineage of development. The remainder of this paper will focus on a discussion of the different adult populations of stem cells that have been employed as companion or supportive cells for islets transplants and conclude with a brief description of research utilizing stem cells in an attempt to generate IPCs. The different roles played by these cells are summarized in Table 1.

## 4. Stem Cells as Companion Cells

### 4.1. Mesenchymal Stem Cells

Among the most studied of adult stem cells as companion or supportive cells for islet transplantation are mesenchymal stem cells (MSCs). MSCs are multipotent progenitor cells found in the perivascular spaces of many adult tissues. These cells have the capacity for self-renewal. MSCs may be able to differentiate into mesodermal and potentially ectodermal and endodermal lineages, but the ability of these cells to differentiate into all three lineages remains somewhat controversial [76]. The multipotent, immunomodulatory, and regenerative properties of these cells have inspired applications in models of tissue injury and immune diseases, ranging from increased neurogenesis in rats to inhibition of proinflammatory cytokines in murine acute lung injury models [76]. 

In preclinical studies, cotransplantation of islets and MSCs has emerged as a promising tool to improve graft survival. Early studies focused on the effects of bone-marrow derived MSCs and the benefits of this cell population on transplanted islet function have been demonstrated repeatedly through *in vivo* experiments in rodents and primates [77]. Cotransplantation with syngeneic MSCs results in a lower *β* cell requirement for normoglycemia, with observed improvements in glucose tolerance and prolonged viability of allogeneic islet transplants in mice [31, 39–41]. In diabetic cynomolgus monkeys at 1 month after transplantation, the combination of MSCs with islets confers prolonged graft function with significantly increased C-peptide levels compared to islets transplanted with nonspecific bone marrow cells [32]. Because of their adhesive properties, MSCs have also been shown to coat islets in a coculture system. This characteristic provides a potential model for transplantation that may improve interactions between the cell types after engraftment [78]. 

Numerous studies have been undertaken to investigate the mechanisms behind the beneficial effects of bone marrow-derived MSCs. One important contribution of MSCs appears to be related to their immunomodulatory capabilities. MSC administration in mice with allogeneic islet grafts was associated with decreased delayed-type hypersensitivity via cleavage of CD25 from the T cell surface. This effect limited T cell activation and prolonged graft survival [31]. Additional MSC dosing was associated with reversal of acute rejection of allogeneic transplants in monkeys [32]. These properties are mediated via production of multiple factors that collectively act to suppress T cell proliferation and function, dendritic cell maturation, and natural killer-cell proliferation. MSC-derived factors act on these immune cells to decrease secretion of proinflammatory cytokines including interferon gamma, granulocyte-macrophage colony-stimulating factor, tumor necrosis factor-*α*, and monocyte chemoattractant protein-1 [31, 33–35]. MSCs also act to induce regulatory T cells and the generation of anti-inflammatory cytokines like IL-10, modulate neutrophil function, and B cell function and differentiation [35, 37]. Collectively, these effects create a shift away from antigen-specific cytotoxicity and inflammation [31, 36–38, 42, 57]. 

Another component to the advantageous effects of MSCs is their contribution to establishing a vascular network for new islet grafts. Compared to islets transplanted alone, multiple studies have demonstrated that mice transplanted with bone marrow cells or bone marrow-derived MSCs combined with islets had a significant increase in peri-islet vessel number, with a higher capillary to *β* cell ratio observed postoperatively [39–41]. In cotransplantation models, the development of new vessels in hybrid grafts was observed earlier [39]. This earlier and more pronounced increase in capillary density seems to occur secondary to secretion of multiple proangiogenic factors, including vascular endothelial growth factor (VEGF), interleukin 6(IL-6), interleukin 8(IL-8), hepatocyte growth factor (HGF), TGF-*β* (transforming growth factor-*β*), and platelet-derived growth factor [39–44]. MSC secretion of matrix metalloproteinases is also thought to initiate degradation of the preexisting extracellular matrix, allowing endothelial cells to migrate into islets. This consequence is evidenced by increased vascular sprout development and endothelial cell migration into the surrounding matrix that occurs *in vitro* when MSCs are combined with human islet-endothelial cell composite preparations compared to islets combined with only endothelial cells [31, 45]. 

In addition to proangiogenic effects, MSCs also have potent antiapoptotic effects that protect islets from the hypoxia and inflammatory destruction which occurs as a result of the isolation and transplantation process. In an *in vitro* model of islet hypoxia and reoxygenation, rat islets cocultured with bone marrow MSCs had increased expression of protective hypoxia-induced genes, along with decreased apoptotic rates, and improved glucose-stimulated insulin secretion when compared to islets cultured alone [46]. Cocultured islets also had an increased ATP/ADP ratio leading to improved glucose-stimulated insulin release [42, 47, 79]. Rat islets treated with streptozotocin to mimic peritransplantation inflammation had decreased apoptosis and increased glucose stimulated insulin secretion when indirectly cocultured with MSCs [48]. *In vivo* benefits of these cells on early islet death from the isolation process are demonstrated by improved blood glucose values in diabetic mice receiving a marginal mass of human islets that were cultured in MSC media for 48 hours before transplantation. This improvement was noted when compared to results obtained from transplantation of islets that had undergone more typical isolation procedures [47]. 

Many of the effects of MSCs appear to be mediated via secretion of paracrine factors, including HGF, TGF-*β*, IL-6, VEGF A, and platelet-derived growth factor [42]. The importance of this influence is supported by a decrease in benefits on islet survival and vessel development observed when human islets are cocultured with bone marrow cells and antibodies that selectively deplete these paracrine factors [80]. Direct cell contact between the MSCs and islets may also play a role, however, as immunomodulatory effects and IL-10 production are decreased *in vitro* when islets are separated from MSCs by a permeable membrane [38]. 

Concurrent transplantation of islets with MSCs also has advantageous effects on islet remodeling and structure that may lead to improved insulin secretion as well as improved intraislet paracrine communication between *β* cells and other islet endocrine cells [81–84]. Immunostaining reveals in mice, islets transplanted with MSCs develop graft morphology characteristic of native islet architecture, versus a more diffuse distribution of *α* cells and *δ* cells in grafts containing only islets [49]. After 6 months of coculture with MSCs, human islets maintained a three-dimensional shape that contained endothelial cells compared to development of a monolayer from islets cultured alone. Reverse transcriptase-PCR also revealed improvements in glucagon expression in cocultured islets [80]. Further *in vivo* data on the impact of MSC companion cells on the function of other intraislet hormone-producing cells is needed to characterize the significance of this effect.

### 4.2. Adipose-Derived Stem Cells

More recent studies have demonstrated the potential of additional postnatal organs to function as sources of MSCs [77]. Adipose tissue has emerged as a promising origin of these adult stem cells with regenerative capacity [85]. MSCs derived from adipose tissue (ASCs) are obtained from the adipose stromal vascular fraction, a population of cells obtained after enzymatic dissociation of adipose depots followed by density separation from adipocytes [86]. ASCs may also be able to differentiate into mesodermal and potentially ectodermal and endodermal lineages, but again the ability of the cells to differentiate into all lineages is somewhat controversial [87]. While ASCs are functionally similar to bone marrow MSCs, they are more easily accessible with minimal risk to the patient. Adipose also yields a greater number of stem cells per gram of tissue than bone marrow [85, 88]. This accessibility is especially attractive as patients could easily provide their own populations of cells. 

ASCs exhibit a number of potential characteristics and effects that are similar to MSCs and benefit islet grafts comparably [85]. Combined transplantation of ASCs with a marginal islet mass resulted in prolonged graft survival and glucose tolerance similar to that observed when using significantly higher numbers of islets. Hybrid grafts had a well-preserved islet structure compared to those transplanted with islets alone. These hybrid islets also had decreased presence of CD4+ and CD8+ cells, reflecting an anti-inflammatory effect [57]. Pretreatment of ASCs with a mixture of molecules to increase paracrine factor secretion, followed by coculture with islets, then combined transplantation of islets and ASCs has been shown to further improve graft function [89].

While research into the application of ASCs to diabetic models is ongoing, ASCs have been studied in several other injury and disease models. Studies in mice with proximal femoral artery ligation and subsequent hindlimb ischemia have demonstrated the pro-angiogenic influence of ASC administration [90]. *In vitro* studies suggest the etiology is a combination of differentiation and direct incorporation of ASCs into vascular structures combined with secretion of angiogenic and antiapoptotic growth factors [55, 90]. These specifically include VEGF, HGF, basic fibroblast growth factor (bFGF), granulocyte-macrophage colony-stimulating factor (GM-CSF), and TGF-*β* [37, 55, 56]. ASC-hybrid grafts demonstrate an increased presence of endothelial cells, which appear to be differentiated from ASCs [57].

### 4.3. Endothelial Progenitor Cells

Possible benefits on vasculogenesis have generated interest in endothelial progenitor cells (EPCs), which promote angiogenesis at sites of hypoxia or injury and can be obtained from bone marrow, cord blood, vessel walls, or peripheral blood [91]. The use of EPCs in ischemic injury models has previously been undertaken [92, 93]. In a rat model of myocardial infarction, EPC transplantation was associated with improved ventricular function [94]. Microvesicles derived from EPCs enhanced limb perfusion in mice with femoral artery ligation [95]. Effects are mediated via direct differentiation into new vessels and possibly through secretion of paracrine factors that support the growth of new vasculature [96, 97]. Interestingly, vessel formation after islet transplantation is thought to be unstable secondary to an inability to attract sufficient host mural cells [55]. However, generation of a more stable vascular network has been achieved by cotransplantation of endothelial cells with ASCs. In this context, ASCs are able to function similarly to pericytes, which are cells that line vessel walls and support vasculature. This role is supported by frequent ASC expression of pericyte surface markers and the periendothelial location of ASCs in adipose tissue *in vivo*. Through paracrine interaction, endothelial cells promote mitosis and chemoattraction of ASCs, while ASCs promote endothelial cell survival and migration [55]. The potential for use of this cell mixture in islet transplants is supported by the development of a vascular network with clusters of insulin-positive cells when islets were combined with subcutaneous implants in mice [61]. 

### 4.4. Neural Crest Stem Cells

The utility of other adult stem cells as companion cells has also been explored. Islet innervation plays an important role in *β* cell development and function [98, 99]. Disruption of this nerve supply occurs during the isolation and transplantation process. The importance of innervation for islet function led to the hypothesis that neural crest stem cells may be valuable companion cells in islet grafts. *In vitro,* islets have a trophic effect on neural crest migration and promote differentiation into neurons [62]. Neural crest stem cells cotransplanted with islets migrate and associate with the islet cells. Hybrid grafts with neural crest cells and marginal islet mass developed similar *β* cell mass when compared to transplants that began with twice the amount of islets. These hybrid grafts functioned similarly to the islet-only grafts by the one-month time point, with no significant differences observed in glucose tolerance [62, 63]. 

Whether utilized individually or in combination with other supportive cells, the addition of adult stem cells as companions to islet allografts provides a promising avenue to address the limitations afforded by the current transplantation process. While a wealth of preclinical data suggests this approach is feasible with innumerable benefits, further studies in human clinical trials are needed to determine if the myriad of benefits observed in animal models will extend to human islet transplantation strategies. 

## 5. Human Embryonic Stem Cells (hESCs) and the Quest to Generate an Alternative Source of Insulin-Producing Cells 

The prospect of a limitless and renewable replacement for *β* cells has inspired multiple investigations involving human embryonic stem cells (hESCs) [64–66]. By mimicking steps in the typical development of pancreatic endocrine cells, early studies have been able to induce differentiation of hESCs into cells that express insulin and other *β* cell markers. However, low yields of functionally immature, ineffective cells rendered clinical utilization of this approach impractical as these cells had vastly decreased amounts of insulin compared to normal islets. Further, these early cells were unable to correct hyperglycemia in mice rendered diabetic by streptozotocin [67, 68]. More recent advancements in the understanding of embryonic *β* cell development have resulted in more successful differentiation protocols. These approaches generate higher yields of cells expressing markers of pancreatic endoderm. Once transplanted, these cells differentiate into functional endocrine cells [69–71]. The microenvironment of various graft sites surrounding the transplanted cells may also have important effects on subsequent differentiation [70]. Diabetic mice transplanted with hESCs treated under these newer protocols experience sustained correction of their hyperglycemia and have comparable insulin and C-peptide levels to mice transplanted with large numbers of islets. These mice had immediate recurrence of hyperglycemia upon removal of the grafts [69, 72]. These findings suggest an *in vivo* contribution to the terminal differentiation of hESCs into IPCs. Despite these advances, there is little known about the *in vivo* factors or conditions needed for these terminal maturation steps. This remains an area of intense study. 

Exploration into the possibility of reprogramming somatic cells into cells that resemble hESCs has also resulted in promising outcomes. Although autoimmunity would still present a challenge, this prospect is especially enticing given that patient-specific *β* cells could be generated, circumventing the need for immunosuppression to prevent rejection. Social and ethical objections to use of hESCs would also be avoided. These induced pluripotent stem cells (iPSCs) could ideally be directed down a pancreatic endocrine developmental program and then be used to produce insulin producing cells. While this field has not yet advanced as far as the human embryonic stem cell field, several studies utilizing these protocols show the generation of IPCs that express insulin and some other markers of mature *β* cells [73, 74, 100]. *In vivo*, murine fibroblasts have been utilized to generate iPSCs, then differentiated into IPCs that improve hyperglycemia when transplanted via the portal vein in mice treated with streptozotocin [75]. IPCs generated from rhesus monkey fibroblasts using similar techniques were able to normalize hyperglycemia in about half of diabetic mice receiving renal subcapsular transplants [50]. Early studies have also utilized viral vectors to infect somatic cells and induce expression of transcription factors important for pluripotency. The risk of mutagenesis associated with genomic integration when using this approach made it unsuitable for therapeutic use [101, 102]. Newer approaches continue to be developed that employ alternative methods of gene delivery, including direct delivery of reprogramming proteins that are capable of penetrating the cell membrane without a viral vector, or bacterial delivery of these nuclear proteins [103, 104]. 

Despite some promising results of investigations using hESCs and iPSCs to generate IPCs, several other limitations exist that are unique to these cell populations. In addition to the incomplete differentiation, the recombinant proteins required for the differentiation process are extremely expensive. Current research is exploring chemical compounds that could replace these proteins in protocols, while providing more easily regulated and efficient processes of guiding differentiation. Examples include histone deacetylase inhibitors, (−)-indolactam V, and a cocktail consisting of inhibitors of transforming growth factor-*β* (TGF*β*) and extracellular signal-related kinase pathways and thiazovivin [105–107]. The usage of reproducible large-scale systems to generate populations of progenitor cells will be necessary for clinical feasibility [108]. 

Safety concerns have also arisen regarding teratoma formation from undifferentiated cells [69, 109]. The incidence of teratoma may be decreased by more effective purification methods, avoiding transplantation of other pluripotent cells, or by insertion of pancreatic transcription factor genes that limit pluripotency [72, 109]. However, the ideal approach remains to be elucidated. Another potential complication, the differentiation of pancreatic progenitor cells into acinar-derived dilated ducts and cysts that could impinge on functional IPCs, has also been recently described [108].

Some have explored the possibility of forcing adult stem cells that could otherwise be used as supportive cells in transplantation strategies towards a *β* cell lineage *in vitro*. These cells may then be able to directly contribute to islet graft success through differentiation into IPCs [110]. This approach could still take advantage of patient-specific cells, avoid a need for immunosuppression, and circumvent some of the complications that arise with pluripotent stem cell usage. Importantly, this approach should theoretically reduce teratoma formation. Differentiation of bone marrow MSCs has been induced *in vitro* with high glucose and nicotinamide-enriched culture mediums. The resulting IPCs were able to temporarily control hyperglycemia in streptozotocin-induced diabetic rats [51, 52]. Normoglycemia was also demonstrated in streptozotocin-treated mice after receiving MSCs that had been differentiated into IPCs from skin fibroblasts using a 3-stage protocol [53]. IPCs generated from placental and umbilical cord MSCs have similarly been reported to decrease hyperglycemia in diabetic mice [50, 54]. 

Conflicting *in vivo* evidence exists for IPC development from MSCs that have not undergone a differentiation protocol. In mice transplanted with islets and bone marrow derived MSCs, an increase in pancreatic and duodenal homeobox gene (PDX-1) positive cells was noted in bone-marrow cells in the postoperative period. This may have reflected an increase in islet cell precursors, although no insulin positive cells developed over the course of the experiment [39]. Other *in vivo* studies have had negative results, with no evidence of MSC-derived *β* cells observed in murine pancreatic injury or transplantation models, despite improved islet graft function [43, 81]. These studies reflect that the majority of MSC effect on *β* cell regeneration likely occurs through augmentation of endogenous cell survival or regeneration. 

Similar to bone marrow MSCs, ASCs are capable of differentiation into primitive IPCs *in vitro* under certain culture conditions [58, 59]. ASCs differentiated into islet-like aggregates were able to produce detectable C-peptide and improve hyperglycemia in diabetic mice undergoing transplantation with the cells. Interestingly, these improvements were similar to those seen when transplanting undifferentiated ASCs, suggesting that more work is still needed to identify mechanisms of improvement [60]. Analogous to MSCs, much of the evidence regarding ASC effects on islet replacement points to their role as supportive cells.

Although much progress continues towards the goal of creating a renewable source of engineered *β* cells from stem cells, further research will be necessary for the realization of this goal in humans. Still, this remains an area of intense study as multiple high-profile groups within academia and industry work towards creating insulin producing cells from embryonic, induced pluripotent, or adult stem cells. 

## 6. Human Clinical Trials

 Although no clinical human trials have been published that employ stem cells in islet transplantation strategies, they are beginning to be employed in other ways. Recently, patients with T1DM whose serum lymphocytes were “educated” by multipotent human cord blood stem cells demonstrated a progressive improvement in fasting and stimulated C-peptide levels up to 40 weeks after treatment. “Education” was performed by removing the cells from peripheral blood and returning them to the circulation after stem cell exposure. Patients receiving this novel treatment exhibited a significant increase in regulatory T cells and TGF-*β*1, reflecting immune modulation as an explanation for the improved *β* cell function [111]. Even with clear differences in the treatment approach, these results provide promise for a future role of stem cells in islet transplants for T1DM in humans. 

 Despite the lack of published human clinical trials, a search of registered clinical trials (http://clinicaltrials.gov/) at the time of this paper revealed 15 active studies involving stem cell treatments for TIDM. Thirteen studies involved infusion of stem cells (mostly autologous MSCs), while one study used a stem cell “educator” as outlined above. Only one study planned to evaluate cotransplantation of islets with MSCs. The trials listed appear to be of varying quality, and many factors regarding the administration of these cells will need to be carefully and rigorously studied. For instance, *in vitro* exposure of bone marrow MSCs to human blood can actually trigger the IBMIR. Markers suggestive of a mild IBMIR were noted upon retrospective review of humans receiving therapeutic MSC infusions for complications related to prior hematopoietic stem cell transplants. This effect appears to be dependent on multiple variables including the donor, dose of stem cells, and number of cell passages. Further study will be necessary to elucidate the ideal use of parameters to minimize the risks of IBMIR while maximizing other benefits offered by MSCs [112]. Until further published data is available, physicians should carefully counsel patients who may be eager or desperate for novel treatments for T1DM against “stem cell tourism” or enrollment in experimental protocols without a thorough review of the quality of ongoing studies.

## 7. Conclusion

Much progress remains to be achieved in the field of islet transplantation in order for this procedure to offer a suitable alternative to exogenous insulin replacement. Stem cells provide an effective aid to address immune-mediated graft dysfunction and poor vascular supply, while supporting *β* cell growth and development and inhibiting apoptosis. Although much work is needed in the field, stem cells may also serve as a renewable source of insulin producing cells. Through these diverse roles, stem cells may provide the key to bridging the gap between the current status of transplant outcomes and a viable long-term solution to insulin deficiency. In order to move this field forward, human data will be necessary to provide confirmation of preclinical studies and provide further characterization of the therapeutic benefits offered by stem cells. 

## Figures and Tables

**Table 1:** Described roles of stem cells in islet transplantation.

Stem cell	Function	Mechanism
Mesenchymal stem cells	Immunomodulation	Decreased activation and proliferation of T cells, dendritic cells, and NK cells, thereby decreasing secretion of inflammatory cytokines including interferon gamma, granulocyte-macrophage colony-stimulating factor, tumor necrosis factor-*α*, and monocyte chemoattractant protein-1 [31–36] Induction of regulatory T cell activation and IL-10 production [35, 37, 38] Modulation of neutrophil and B cell function and differentiation [35, 37]
	Establishment of graft vascular network	Secretion of angiogenic paracrine factors VEGF, IL-6, IL-8, HGF, PDGF, and TGF-*β* [39–44] Secretion of matrix metalloproteinases [31, 45]
	Antiapoptotic	Secretion of paracrine factors including HGF, IL-6, and TGF-*β* resulting in increased expression of genes protective against hypoxia [46–48]
	Improved *β* cell architecture	Unknown [49]
	*β* cell replacement	Differentiation into insulin-producing cells [50–54]

Adipose-derived stem cells	Establishment of graft vascular network	Secretion of angiogenic paracrine factors VEGF, HGF, bFGF, GMCSF, and TGF-*β* [37, 55, 56] Differentiation into endothelial cells [57]
	Antiapoptotic	Secretion of antiapoptotic growth factors, including HGF, GM-CSF, and TGF-*β* [56]
	*β* cell replacement	Differentiation into insulin producing cells [58–60]

Adipose-derived stem cells + endothelial progenitor cells	Establishment a vascular network within grafts	Direct differentiation into vasculature and pericytes [61] Release of paracrine factors [61]

Neural crest stem cells	Improved graft innervation	Neuronal differentiation [62, 63]

Human embryonic stem cells	*β* cell replacement	Differentiation into insulin producing cells [64–72]

Induced pluripotent stem cells	*β* cell replacement	Differentiation into insulin producing cells [73–75]

## Abbreviations

T1DM: Type 1 Diabetes Mellitus
IBMIR: Instant blood-mediated inflammatory reaction
IPC: Insulin producing cell
hESC: Human embryonic stem cell
iPSC: Induced pluripotent stem cell
IDE: Induce definitive endoderm
MSC: Mesenchymal stem cell
ASC: Adipocyte-derived mesenchymal stem cell
PDX-1: Pancreatic and duodenal homeobox gene
TGF-*β*: Transforming growth factor-*β*
VEGF: Vascular endothelial growth factor
HGF: Hepatocyte growth factor
TIMP-1: Tissue inhibitor of metalloproteinase 1
bFGF: Basic fibroblast growth factor
GM-CSF: Granulocyte-macrophage colony stimulating factor
IL-6: Interleukin 6
IL-8: Interleukin 8.
